# Supplementary material for: Patterns of Intron Gain and Loss in Fungi
Source: PLoS Biol. 2004 Nov 30;2(12):e422. doi: 10.1371/journal.pbio.0020422 (PMC532390; doi:10.1371/journal.pbio.0020422)
Supplement: Table S1 — Also available at http://genes.mit.edu/NielsenEtAl/. (4.3 MB ZIP). [file pbio.0020422.st001.zip › NielsenEtAl/html/711.html]

AN8990.1.NCU05776.1.MG01385.1.FG06473.1


```
 CLUSTAL W (1.82) Multiple Sequence Alignments - Introns Inserted


Sequence 1: MG01385.1	609 aa
Sequence 2: FG06473.1	527 aa
Sequence 3: NCU05776.1	628 aa
Sequence 4: AN8990.1	542 aa
Alignment Length: 650 aa
Number Identitical Residues: 248 aa
Alignment Score (without introns) 13134


MG01385.1 	METAARVS~DGEAKC--VAV1LRFEIGGSSARNT------PPPTALSYVITGPYLWRWVP
NCU05776.1	MEAAAAPS0TTFARLDGNPV~LSDSVPPAATCNTGHRRTEPASTNICIDVTGSITFCPVP
FG06473.1 	--------~-----------~-------------------------------------MS
AN8990.1  	--------~-----------~-------------------------------------MS
          	                                                          :.

MG01385.1 	SHSCGITR2QTVQQQHKMSQKQVTSDDSLAGNGFATGSQARNDSNKDLAGMHRSHSGMDS
NCU05776.1	PLQLLLFC~AMSTTAAKASGS-----------------YAAGPPDILRPRSHDLGGEVAS
FG06473.1 	KHDSGSTS~TGAMEQPAIDQK------------------PRRNSK---------ASGSGN
AN8990.1  	TPKDTPWA~PATLNESAIETG-------------------------------------SD
          	  .               .                                        .

MG01385.1 	NDSANQVLESLGYKPELQRNRSTWQVAFMSFVLASIPYGLATTMFYPLIGGGPVNIIWGW
NCU05776.1	ASDGDDLLNALGYKAELARTRSTWHVAFMSFVLASIPYGLATTLYYPLQNGGPVVVIWGW
FG06473.1 	PDAADQLLENLGYKAELSRNRSTFQVAFMSFVLASIPYGLATTLAYPLIGGGPVNVIWGW
AN8990.1  	KDGANAALEHLGYTPELSRNRSTWNVVFMSFILASVPYGLSTTYTYALAGGGPTNIIWGW
          	 . .:  *: ***..** *.***::*.****:***:****:**  *.* .***. :****

MG01385.1 	VLVSLIIICVAASLGEITSVYPTAGG~VYYQAFMLADPSWRRAASWICGWLYVVGNITIT
NCU05776.1	VIISAIILCVAASLGEITSVYPTAGG~VYYQTFMIAPAKYRRVSAYICGWAYVVGNITIT
FG06473.1 	LAVSLIIVCVAASLGEITSVYPTAGG1VYYQAFMLSPPRWRRIASWICGWLYIVGNITIT
AN8990.1  	VLVSLIILCVAASLAEITSVFPTAGG~VYYQTFVLSPIWCRRISSWVCGWAYVTGQIMIT
          	: :* **:******.*****:***** ****:*:::    ** ::::*** *:.*:* **

MG01385.1 	LAVNFGTTLFLVACINVFKYDVDGVPTGIFE-GQPYQVFLIFLGLTLFCNAVSSLGNKWL
NCU05776.1	LAVNFGTALFFVACVNVFQNDDG---SDIWQ-AETYQIFLVFLAITLLCNAISALGNKIL
FG06473.1 	LAVNFGTALFIVSCVNVFESSPG---VGVMS-GEAYQVFLVFLGLTFLCNAISALGNKYL
AN8990.1  	LAVNFGSAQFFIACLNVFEDENG---VGLTDSWEAWHTYVIFLAITLVSHAIPAFGNRWL
          	******:: *:::*:***: . .    .: .: :.:: :::**.:*:..:*:.::**: *

MG01385.1 	PILD0TAAIFWTFAGVIAIMVTVLALAKAGRRDAAFVFGHFETNSGWPAGWSFCVGLLHA
NCU05776.1	PLLD0TFAIVWTFVGLICILVTILVVAKEGRHSGAYAFGHFEPTSGWPKGWAFCVGLLHA
FG06473.1 	PWID0TAAVFWTFAGVIAIVVCVLAMAKEGRRDAAYVFGHFEANSGWPKGWSFCVGLLHA
AN8990.1  	PWLE0SFAIFWTLAGVVAIDICLLVIAKNGRRSAKWVFGHFEPQTGWPDGWSFCIGLLQA
          	* :: : *:.**:.*::.* : :*.:** **:.. :.*****. :*** **:**:***:*

MG01385.1 	GYATSSTGMIIS2MCEEVRMPSTQVPKAMVATVVLNTFAGLLFMIPLVFVLPDIQYLIGL
NCU05776.1	GYATSSTGMIIS~MCEEVQRPATQVPKAMVITIVINAIGGLVFMIPLMFVLPDIAMLVAL
FG06473.1 	AYATSSTGMIIS2MCEEVQNPSVQVPKAMVATIFINTFAGLLFIIPLMFVLPDLQQVI--
AN8990.1  	AYATSSTGMIIT2MCEEVRQPALQVPKAMVGTIVINLIAGLIFLVPVCFVMPDLAELAAL
          	.**********: *****: *: ******* *:.:* :.**:*::*: **:**:  : . 

MG01385.1 	ASGQPVPEIILAAVGEPGAAIALLMPLLVLAIICGIGCTTAASRCTWAFARDGAIPGAQW
NCU05776.1	PSGQPVPTILKSAVGSSVGAICLLIPLMVLGILCGTACTTAASRCTWAFARDGAIPGARW
FG06473.1 	LSAQPVPFIIKSAVGSSGGAFGLLFPLIILAIICGIGCTTATSRCTWAFARDGAIPGAKW
AN8990.1  	ASGQPVPSIFKSAIGNSVGTFLLLLPLIILGLICGIGCVTATSRCTWAFARDGAIPGSRW
          	 *.**** *: :*:*.. .:: **:**::*.::** .*.**:***************::*

MG01385.1 	WRVINEKLDVPLNAMMLSMAVQIILGLIYFGSSAAFNAFSGVGVICLTASYATPIAINLF
NCU05776.1	WKEVHTGLDLPLNAMMLSMVIQILLGVIYFGSSAAFNAFSGVGVICLTISYAVPIAVSMI
FG06473.1 	WSKVNTSLDVPLNAMMLSMVVQIILGVIYFGSSAAFNAFSGVGVICLTASYATPIAISLA
AN8990.1  	WRTVNKKLDVPLNSMMLGMVIELLIGLIYFGSSAAYNAFSGVGVILLTLSYACPIAVSLL
          	*  ::  **:***:***.*.:::::*:********:********* ** *** ***:.: 

MG01385.1 	KGRKA-TANAKFQLGRMGVFCNIVALA~WSALAMPLFCMPSYLPVTPETVNYAPVVFVAA
NCU05776.1	EKRKH-VIGAKFPLGKLGWFCNIVALA1WSFFAVPLFCMPAYIPVSAGTVNYAPVVFVGF
FG06473.1 	TGRKQ-VKTGKFYLGKFGAVANVIAIA1WSLLAMPLFCMPSMIPVTPETVNYAPVVFVFA
AN8990.1  	LRRREDIKHGSFDLGALGLFCNIVALA1WTVLVIPLFNFPSYMSVTLETMNYACVVFVGI
          	  *: .   ..* ** :* ..*::*:* *: :.:*** :*: :.*:  *:*** ****  

MG01385.1 	TIVSGVWYIVWGRENYAGPPVQEEYN-------------------------------
NCU05776.1	TSVSLAWYITWGHKNYAGPPTEQVGEDVATMTTGAASAIPGDIKKPVPVEGSLGKKD
FG06473.1 	CLVSGIWYWAWGHKNYAGPPTNED---------------------------------
AN8990.1  	IVISAVWYWIWGYKNYAGPPTDAIDPEADHPAGSSPVQVAKDIQR------------
          	  :*  **  ** :******.:    .    : .:.   . . .
```
